# Design and Synthesis of P(AAm-co-NaAMPS)-Alginate-Xanthan Hydrogels and the Study of Their Mechanical and Rheological Properties in Artificial Vascular Graft Applications

**DOI:** 10.3390/gels10050319

**Published:** 2024-05-07

**Authors:** Zhutong Li, Joshua Giarto, Jue Zhang, Jinsu Gim, Edward Chen, Eduardo Enriquez, Lauren Jafuta, Esha Mahalingam, Lih-Sheng Turng

**Affiliations:** 1 Department of Mechanical Engineering, University of Wisconsin-Madison, Madison, WI 53706, USA; zli572@wisc.edu (Z.L.); edchen@wisc.edu (E.C.); edenriquez96@gmail.com (E.E.); ljafuta@wisc.edu (L.J.); 2 Wisconsin Institute for Discovery, University of Wisconsin-Madison, Madison, WI 53715, USA; jgiarto@wisc.edu (J.G.); emahalingam@wisc.edu (E.M.); 3 Department of Biomedical Engineering, University of Wisconsin-Madison, Madison WI 53706, USA; 4 Morgridge Institute for Research, University of Wisconsin-Madison, Madison, WI 53715, USA; juzhang@morgridge.org; 5 Dongnam Division, Korea Institute of Industrial Technology (KITECH), Jinju 52845, Republic of Korea; jgim@kitech.re.kr; 6 College of Letters and Science, University of Wisconsin-Madison, Madison, WI 53706, USA

**Keywords:** hydrogel, vascular graft, PNaAMPS, PAAm, mechanical and rheological properties

## Abstract

Cardiovascular diseases (CVDs) are the number one cause of mortality among non-communicable diseases worldwide. Expanded polytetrafluoroethylene (ePTFE) is a widely used material for making artificial vascular grafts to treat CVDs; however, its application in small-diameter vascular grafts is limited by the issues of thrombosis formation and intimal hyperplasia. This paper presents a novel approach that integrates a hydrogel layer on the lumen of ePTFE vascular grafts through mechanical interlocking to efficiently facilitate endothelialization and alleviate thrombosis and restenosis problems. This study investigated how various gel synthesis variables, including N,N’-Methylenebisacrylamide (MBAA), sodium alginate, and calcium sulfate (CaSO_4_), influence the mechanical and rheological properties of P(AAm-co-NaAMPS)-alginate-xanthan hydrogels intended for vascular graft applications. The findings obtained can provide valuable guidance for crafting hydrogels suitable for artificial vascular graft fabrication. The increased sodium alginate content leads to increased equilibrium swelling ratios, greater viscosity in hydrogel precursor solutions, and reduced transparency. Adding more CaSO_4_ decreases the swelling ratio of a hydrogel system, which offsets the increased swelling ratio caused by alginate. Increased MBAA in the hydrogel system enhances both the shear modulus and Young’s modulus while reducing the transparency of the hydrogel system and the pore size of freeze-dried samples. Overall, Hydrogel (6A12M) with 2.58 mg/mL CaSO_4_ was the optimal candidate for ePTFE–hydrogel vascular graft applications due to its smallest pore size, highest shear storage modulus and Young’s modulus, smallest swelling ratio, and a desirable precursor solution viscosity that facilitates fabrication.

## 1. Introduction

Cardiovascular diseases (CVDs) are the foremost cause of mortality among non-communicable diseases globally [1], with CVDs accounting for 19.05 million deaths worldwide in 2020 [2,3]. The anticipated rise in fatalities due to CVDs in the future renders it an urgent global issue to be addressed [4].

Bypass surgeries and vascular graft replacement are common surgical approaches for treating CVDs [2,5]. FDA-approved expanded polytetrafluoroethylene (ePTFE) with a porous structure is a type of widely used synthetic material in vascular graft replacement [6,7,8]. However, because of the poor biocompatibility and super-hydrophobic nature of ePTFE, endothelial cells (EC) are hard to adhere to its surface. This limitation can lead to various complications, such as thrombus formation, mechanical property mismatches, and intimal hyperplasia, rendering ePTFE unsuitable for small-diameter vascular grafts (SDVGs) applications [9,10,11,12]. 

There are several physical and chemical modification methods available to address the thrombosis issue of ePTFE vascular grafts. Physical modification methods involve dip-coating or soaking the grafts in biomacromolecules to enhance biocompatibility or reduce thrombogenicity [13,14,15]. However, the dip-coating method can result in easy detachment of biomacromolecules due to blood flow, making it less suitable for long-term vascular graft replacement.

Chemical modification methods entail altering the chemical structure of ePTFE by introducing new functional groups or molecules onto its surface. These methods include the plasma treatment approach to introduce reactive functional groups [16,17], and the covalent bonding modification approach to permanently alter the carbon–fluorine bonds [18,19,20]. However, due to the chemical inertness of ePTFE, the plasma treatment approach is not highly efficient. Additionally, covalent bonding modification requires a challenging modification process using corrosive or chemically active substances to change the carbon–fluorine bond. Moreover, this method can involve toxic chemical reagents, posing potential risks to human health [13].

To compensate for the limitations of ePTFE regarding thrombosis formation, hydrogel with good biocompatibility and endothelialization capabilities can be integrated into the porous ePTFE graft, so that the ePTFE outer layer offers mechanical support while the hydrogel inner layer provides the needed biological function. However, due to the high hydrophobicity and chemically inert nature of ePTFE, achieving a seamless connection of a continuous hydrogel layer to ePTFE without any delamination is challenging.

Currently, there are two existing methods for attaching hydrogel to ePTFE. One method involves employing plasma treatment to alter the ePTFE outer surface or a flat ePTFE surface, generating functional groups like hydroxyl groups, to facilitate bonding with the hydrogel [21,22]. Although this method enables the attachment of a hydrogel to the outer surface of an ePTFE graft or a flat ePTFE film, achieving functionalization of the luminal surface of an ePTFE vascular graft through plasma treatment remains highly challenging. The plasma induced by a common plasma etch system poses a significant obstacle in reaching the lumen of an ePTFE vascular graft, often resulting in unsuccessful functionalization attempts. 

Another method to incorporate a hydrogel layer onto ePTFE is the mechanical interlocking method. It has been reported that the mechanical interlocking method could improve the attachment between a hydrogel and an elastomer [23,24]. To the best of our knowledge, this approach has not yet been utilized in connecting a hydrogel system with ePTFE for the fabrication of an artificial vascular graft. In previous research, we proposed this novel method of attaching a hydrogel layer to the lumen of an ePTFE vascular graft [25]. In this study, we introduce an improved hydrogel formulation that further facilitates the fabrication process and enhances the material’s performance. By mechanically interlocking the well-designed hydrogel to the porous ePTFE vascular graft, a continuous hydrogel layer was connected to the lumen of the ePTFE vascular graft. Through this synergistic approach, the advantages of both the ePTFE’s mechanical stability and the hydrogel’s cytocompatibility and hemocompatibility were realized.

**Figure 1:**
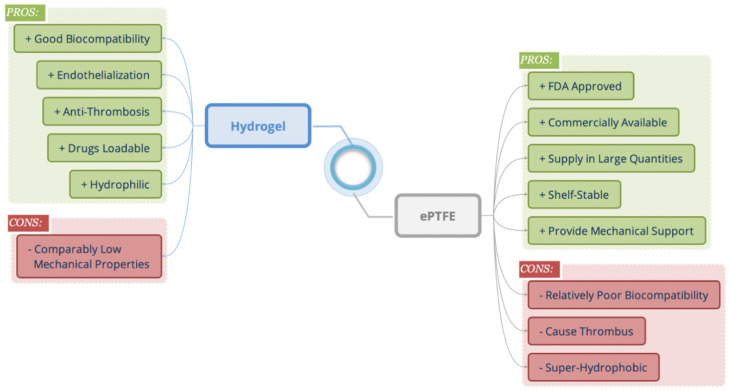
The respective strengths and weaknesses of employing hydrogel and ePTFE in vascular graft applications and the potential synergy of combining them.

The respective advantages and disadvantages of ePTFE and hydrogel and the synergy of combining them in vascular graft applications are shown in Figure 1. The benefits of a well-designed hydrogel include excellent biocompatibility, enhanced endothelialization, anti-thrombosis properties, capacity for drug loading, and hydrophilicity [25]. Furthermore, FDA-approved ePTFE offers advantages such as wide commercial availability, large-scale supply, shelf stability, and provision of mechanical support. By introducing a hydrogel layer on the surface of ePTFE, a vascular graft possessing both robust mechanical properties and favorable biological attributes can be fabricated.

The endothelium, comprising a monolayer of endothelial cells (ECs) within the inner tunica, can release and regulate key molecules, playing an important role in achieving anti-thrombogenic properties in blood vessels [10,26,27,28]. Thus, endothelialization is crucial for preventing thrombosis and restenosis in vascular grafts, ultimately facilitating long-term lumen patency. Our previous study suggests that interpenetrating hydrogels with favorable rheological properties and endothelialization functions are promising candidates for mechanical interlocking with porous and super-hydrophobic ePTFE [25]. This method allows the hydrogels to connect with ePTFE, facilitating the endothelialization process within ePTFE vascular grafts and effectively resolving thrombosis issues associated with plain ePTFE grafts. 

Polyacrylamide (PAAm) is renowned for its favorable rheo-mechanical properties [29,30,31], while poly(2-Acrylamido-2-methyl-1-propanesulfonic acid sodium) (PNaAMPS) offers endothelialization functionality due to its sulfonate groups [25,32,33,34]. Both can be used in the hydrogel system. Based on our prior study, the optimal monomer ratio is AAm/NaAMPS = 40:60, which provided the best endothelialization function, excellent hemocompatibility, a prolonged activated partial thromboplastin time, and desirable rheological properties [25]. In addition to the impact of monomer ratios, the amount of crosslinker also plays a significant role in providing the rheological and biological properties of hydrogels.

To introduce a hydrogel layer on the ePTFE surface, an appropriate range of viscosities of the hydrogel precursor solutions is needed to ensure that the solution can remain in position within the ePTFE porous structure until crosslinking is complete. Insufficient viscosity of the precursor solution could lead to the liquid draining out before the hydrogel is fully crosslinked. Conversely, excessively high viscosity could hamper the injection of the hydrogel precursor solution into the ePTFE porous structure. Hence, considering the proper viscosity of hydrogel precursor solutions is crucial when designing a hydrogel system that is to be attached to the ePTFE vascular graft for endothelialization and anti-thrombosis purposes. Xanthan gum is a polysaccharide with excellent biocompatibility, and it exhibits the ability to offer elevated solution viscosity and maintain gel stability even at very low concentrations [35]. Moreover, it can undergo ionic crosslinking with calcium sulfate [36,37]. Similarly, sodium alginate, another type of natural polysaccharide, can increase the viscosity of hydrogel precursor solutions and provide mechanical energy dissipation function for a hydrogel system through ironic crosslinking [30,38,39,40].

In this study, P(AAm-co-NaAMPS)-alginate-xanthan hydrogels with different compositions of crosslinkers and thickeners were synthesized, and the viscosities of different hydrogel precursor solutions were studied. The impact of each composition on the properties of hydrogels in terms of swelling ratio, transparency, pore size, shear storage modulus, and Young’s modulus of hydrogels was investigated to provide guidance for synthesizing hydrogels suitable for vascular graft applications.

## 2. Results and Discussion

### 2.1. Physical Appearance and Microstructure of Hydrogels

**Figure 2:**
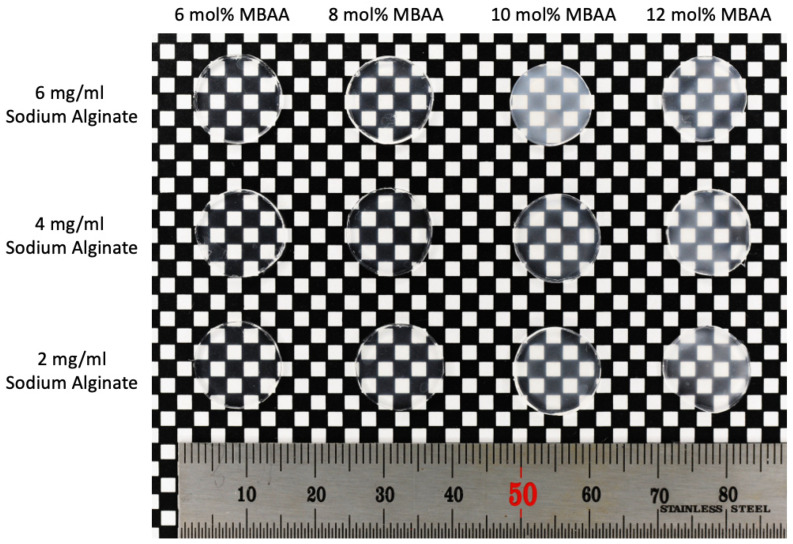
The physical appearance of P(AAm-co-NaAMPS)-alginate-xanthan hydrogels with different amounts of MBAA crosslinker and sodium alginate.

The physical appearance of P(AAm-co-NaAMPS)-alginate-xanthan hydrogels containing varying amounts of MBAA crosslinker and sodium alginate is shown in Figure 2. From bottom to top, there is an increase in sodium alginate concentration in the hydrogel system, and from left to right, the MBAA crosslinker concentration increases. Overall, with an increase in either sodium alginate concentration or MBAA crosslinker concentration, the transparency of the hydrogel decreases. The hydrogel with 10 mol% MBAA and 6 mol% sodium alginate is the exception with the opaquest appearance. The opaqueness of the Hydrogel (6A10M) sample is likely due to the presence of uneven crystallization spots, as shown in its microstructure to be discussed below.

**Figure 3:**
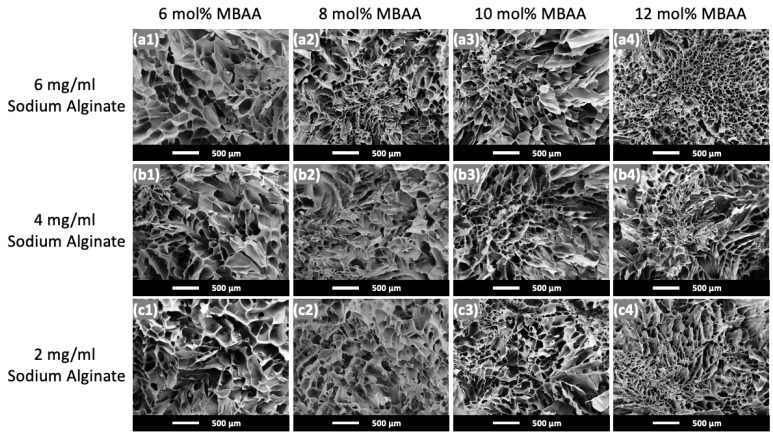
The microstructure of P(AAm-co-NaAMPS)-alginate-xanthan hydrogels with different amounts of MBAA and sodium alginate captured through SEM. (**a1**–**a4**) The microstructure of the Hydrogel (6A6M), Hydrogel (6A8M), Hydrogel (6A10M), and Hydrogel (6A12M) hydrogel systems with 6 mg/mL sodium alginate, respectively. (**b1**–**b4**) The microstructure of the Hydrogel (4A6M), Hydrogel (4A8M), Hydrogel (4A10M), and Hydrogel (4A12M) hydrogel systems with 4 mg/mL sodium alginate, respectively. (**c1**–**c4**) The microstructure of the Hydrogel (2A6M), Hydrogel (2A8M), Hydrogel (2A10M), and Hydrogel (2A12M) hydrogel systems with 2 mg/mL sodium alginate, respectively.

The microstructures of the hydrogels with different amounts of MBAA crosslinker and sodium alginate and after freeze-drying are shown in Figure 3. Same as in Figure 2, the sodium alginate concentration in the hydrogel system increases from bottom to top, and the MBAA crosslinker concentration increases from left to right. 

The average pore size for each hydrogel composition was analyzed by selecting nine random pores within an SEM image, and the results are summarized in Table 1. As the MBAA concentration increases from 6 mol% MBAA to 12 mol% MBAA in Figure 3, there is an overall decreasing trend in pore size. However, Hydrogel (6A10M) is again an exception, displaying less consistent pore sizes and some uneven crystallization spots, which presumably leads to an opaque appearance (cf. Figure 2).

The average pore sizes and the corresponding standard deviations of P(AAm-co-NaAMPS)-alginate-xanthan hydrogels are shown in Table 1. As MBAA concentration increases, there is a general reduction in pore size, except for hydrogels with less than 10 mol% MBAA and with 6 mg/mL sodium alginate. Hydrogel (6A12M), with the highest concentrations of both MBAA and sodium alginate, exhibits the smallest pore size. In comparison, Hydrogel (2A6M), with the lowest concentrations of both MBAA and sodium alginate, has the largest pore size. The amount of MBAA plays an important role in controlling the pore size of hydrogels as it enhances the crosslinking density of P(AAm-co-NaAMPS) with more MBAA crosslinkers added to the hydrogel system. When MBAA increases, the number of covalent bonds connecting polymer chains within the hydrogel network also increases, resulting in smaller pore sizes within a hydrogel network [41].

### 2.2. Physical Appearance and Microstructure of ePTFE–Hydrogel (6A12M) Vascular Graft

**Figure 4:**
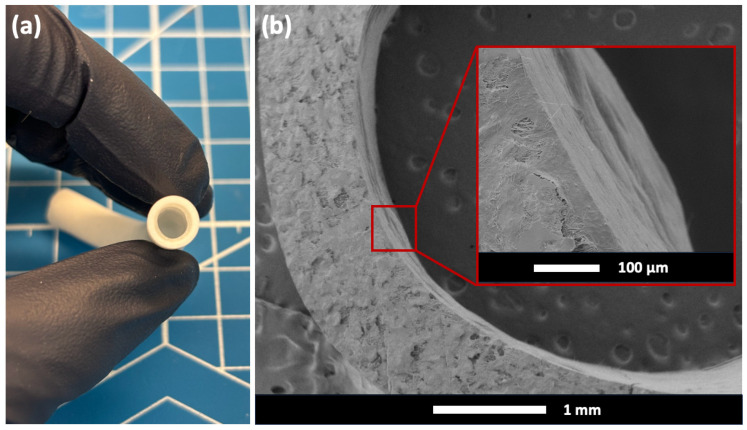
(**a**) Physical appearance of mechanically interlocked ePTFE–Hydrogel (6A12M) vascular graft. (**b**) SEM images showing the cross-section and microstructure of ePTFE–Hydrogel (6A12M) vascular graft.

The physical appearance of the ePTFE–Hydrogel (6A12M) vascular graft is shown in Figure 4a. This photo demonstrates that a continuous thin layer of hydrogel that was well connected to the super-hydrophobic ePTFE graft through mechanical interlocking without any delamination. 

The ePTFE outer layer has an inner diameter of 6 mm and a thickness of 1 mm. The hydrogel inner layer in Figure 4a, with an average thickness of approximately 0.4 mm, is significantly thinner than in our prior work [25]. A thinner and continuous hydrogel layer not only serves as the substrate for endothelial cell attachment but allows for a larger cross-sectional area for blood flow. However, achieving a uniform thickness for the hydrogel inner layer presents a greater challenge with thinner hydrogels than with thicker ones.

The microstructure of the mechanically interlocked ePTFE–Hydrogel (6A12M) vascular graft taken by SEM is shown in Figure 4b. This SEM image illustrates the seamless integration of the crosslinked hydrogel with the porous ePTFE. 

### 2.3. FTIR Investigation of Hydrogels

**Figure 5:**
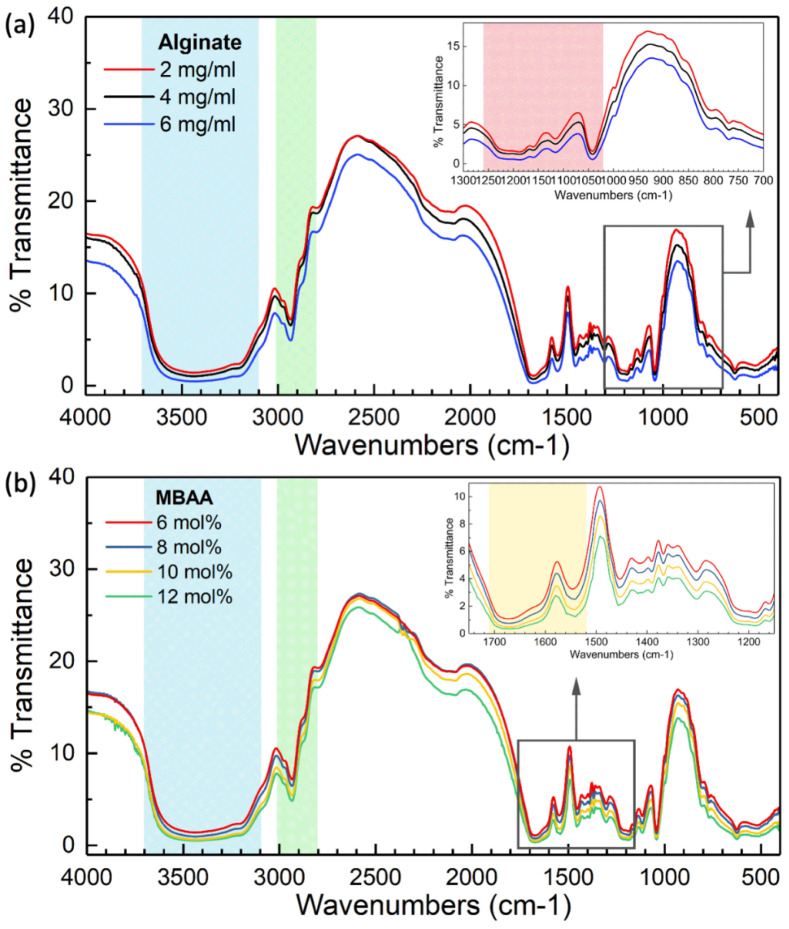
FTIR analysis of P(AAm-co-NaAMPS)-alginate-xanthan hydrogels with different compositions. The blue region represents the stretching of O-H bonds in carboxyl and hydroxyl groups found in xanthan and alginate. The green region corresponds to the vibrations of asymmetrical and symmetrical stretching of CH_3_-, CH_2_-, and CH- groups present in both PAAm and PNaAMPS. (**a**) FTIR results of P(AAm-co-NaAMPS)-alginate-xanthan hydrogels with different amounts of sodium alginate (alginate). The red region indicates the S=O stretching due to the sulfonate group in the hydrogel system. (**b**) FTIR results of P(AAm-co-NaAMPS)-alginate-xanthan hydrogels with different concentrations of MBAA. The yellow region is caused by the amide I band with the C=O stretching vibration as well as the amide II bands associated with the N-H bending vibration.

Figure 5 illustrates the FTIR spectra representing different functional groups found in hydrogels with varied MBAA and alginate contents. In Figure 5a,b, the strong, wide peak from 3700 cm^−1^ to 3100 cm^−1^ in the blue region indicates the stretching of O-H bonds in carboxyl and hydroxyl groups present in xanthan and alginate. The peaks observed between 3000 cm^−1^ and 2800 cm^−1^ in the green region correspond to the vibrations of asymmetrical and symmetrical stretching of CH_3_-, CH_2_-, and CH- groups present in both PAAm and PNaAMPS. The two peaks ranging from 1710 cm^−1^ to 1520 cm^−1^ in the yellow region in Figure 5b are caused by the amide I band with the C=O stretching vibration as well as the amide II bands associated with the N-H bending vibration. The two strong peaks from 1260 cm^−1^ to 1020 cm^−1^ in the red region in Figure 5a indicate the S=O stretching due to the sulfonate group in the hydrogel system. The FTIR results confirm the success of the hydrogel synthesis process. 

### 2.4. Equilibrium Swelling Ratio of Hydrogels

**Figure 6:**
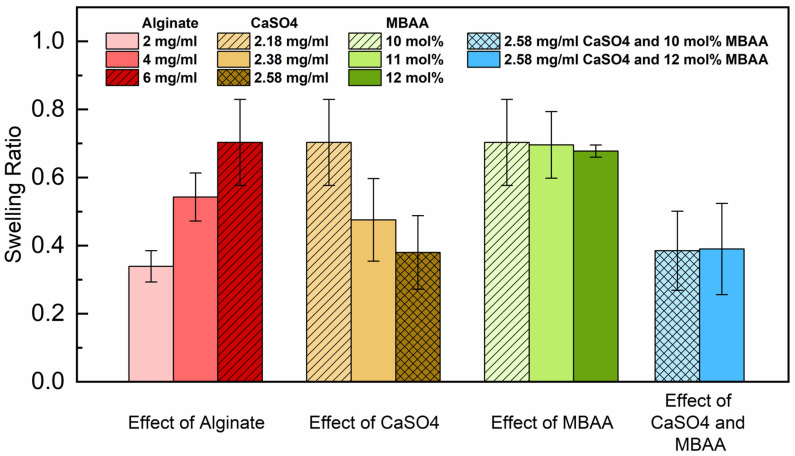
The equilibrium swelling ratio of P(AAm-co-NaAMPS)-alginate-xanthan hydrogels with different amounts of alginate, CaSO_4_, and MBAA. The equilibrium swelling ratio outcome depicted by the dashed lines corresponds to the same hydrogel composition: Hydrogel (6A10M) with 2.18 mg/mL CaSO_4_. The equilibrium swelling ratio result marked with the meshed pattern also corresponds to the same hydrogel composition: Hydrogel (6A12M) with 2.58 mg/mL CaSO_4_.

For hydrogels employed in vascular graft applications, a smaller equilibrium swelling ratio is preferable. The impact of alginate, CaSO_4_, and MBAA on equilibrium swelling ratios of P(AAm-co-NaAMPS)-alginate-xanthan hydrogels was investigated. Figure 6 illustrates the equilibrium swelling ratios of these different types of hydrogels with various compositions.

The red group (first group on the left) in Figure 6 shows that, when maintaining the other components constant, increasing the sodium alginate content from 2 mg/mL to 6 mg/mL led to an increase in the equilibrium swelling ratio of the hydrogels from 0.35 to 0.71. Therefore, with more sodium alginate introduced to the hydrogel system, the swelling ratio of P(AAm-co-NaAMPS)-alginate-xanthan hydrogels also increases.

Furthermore, as indicated by the brown (and second) group in Figure 6, the swelling ratio decreased from 0.71 to 0.38 with the increase in CaSO_4_ concentration from 2.18 mg/mL to 2.58 mg/mL. Hence, increasing the amount of CaSO_4_ leads to a reduction in the swelling ratio of the hydrogel systems. The addition of more CaSO_4_ contributes to counterbalancing the swelling ratio elevation induced by the addition of sodium alginate. This is because a higher amount of CaSO_4_ enhances the ionic crosslinking of sodium alginate in the hydrogel system, decreases the molecular mobility, and restricts the movement of polymer chains, thereby elevating the stability of the hydrogels when immersed in a DPBS solution. The results can also be generalized and applied to other ionically crosslinked hydrogel systems beyond sodium alginate and xanthan. 

Moreover, as shown in the green (third) group in Figure 6, the equilibrium swelling ratio of hydrogels remains approximately at 0.71 with the CaSO_4_ concentration of 2.18 mg/mL, exhibiting minimal change despite the increase in the MBAA crosslinker from 10 mol% to 12 mol%. This indicates that although the concentration of MBAA affects the pore size of the hydrogels in their microstructure as indicated in Figure 3, it has a minimal impact on the equilibrium swelling ratio of P(AAm-co-NaAMPS)-alginate-xanthan hydrogels.

Finally, the blue (and last) group of hydrogel samples have varying MBAA concentrations while maintaining a constant CaSO_4_ content of 2.58 mg/mL. Despite the 2 mol% difference in MBAA content among the hydrogels, a consistent average swelling ratio of around 0.39 persists. These results reaffirm the minimal influence of MBAA on the swelling ratio of hydrogels.

### 2.5. Mechanical Properties of Hydrogels

#### 2.5.1. Rheology Test Results of Hydrogel Precursor Solutions

**Figure 7:**
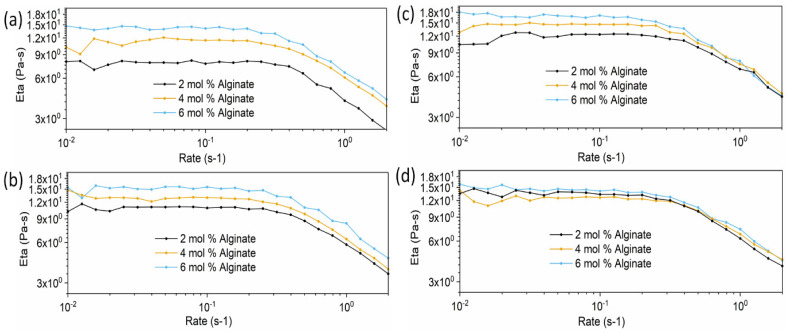
The viscosities of P(AAm-co-NaAMPS)-alginate-xanthan hydrogel precursor solutions with different compositions. The viscosities of hydrogel precursor solutions containing (**a**) 6 mol% MBAA, (**b**) 8 mol% MBAA, (**c**) 10 mol% MBAA, and (**d**) 12 mol% MBAA, respectively.

The viscosities of P(AAm-co-NaAMPS)-alginate-xanthan hydrogel precursor solutions with different compositions are presented in Figure 7. The MBAA concentrations are 6 mol%, 8 mol%, 10 mol%, and 12 mol% for Figure 7a, Figure 7b, Figure 7c, and Figure 7d, respectively.

Specifically, in Figure 7a, at a 6 mol% MBAA concentration, the viscosity of the hydrogel precursor solution is increased when the alginate content is increased from 2 mg/mL to 6 mg/mL. Figure 7b,c demonstrate a consistent trend with Figure 7a as the MBAA concentration increases. However, the extent of viscosity increase diminishes from Figure 7a–d. This indicates that with the increase in MBAA, the influence of alginate on the viscosity change of hydrogel precursor solutions becomes smaller, and the effect of alginate concentration on the viscosity of hydrogel precursor solutions becomes minimal when MBAA reaches 12 mol%.

To prevent the drainage of the hydrogel precursor solution from the porous ePTFE vascular graft before polymerization and crosslinking, a precursor solution with a higher viscosity is preferable. This more viscous precursor solution ensures better retention of the solution within the porous structure of the ePTFE, thereby avoiding drainage before the polymerization and crosslinking processes take place in the oven. Therefore, the hydrogel systems with the maximum alginate concentration of 6 mg/mL are the preferred choice for vascular graft applications.

#### 2.5.2. Rheology Test Results of Crosslinked Hydrogels

**Figure 8:**
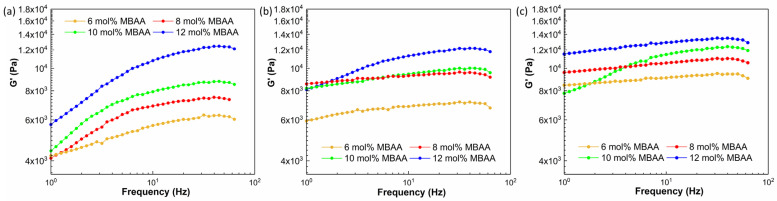
The shear storage modulus of P(AAm-co-NaAMPS)-alginate-xanthan hydrogels with different compositions: (**a**) The shear storage modulus of hydrogels with 2 mg/mL alginate concentration. (**b**) The shear storage modulus of hydrogels with 4 mg/mL alginate. (**c**) The shear storage modulus of hydrogels with 6 mg/mL alginate.

The shear storage moduli of P(AAm-co-NaAMPS)-alginate-xanthan hydrogel samples tested through frequency sweep, parallel-plate rheological tests are shown in Figure 8. As shown in Figure 8a–c, with the increase in MBAA, the shear storage modulus (G’) of hydrogels also increases at frequencies greater than 10 Hz, and the maximum shear storage modulus was attained with a 12 mol% MBAA concentration across all alginate concentrations. 

While the swelling ratio remains relatively unchanged with increasing MBAA content, as shown in Figure 6, the impact of MBAA content on the pore size and shear storage modulus of a hydrogel should not be disregarded. Increased MBAA content leads to greater crosslinking density as well as smaller pore size within a hydrogel system. When the crosslinking density increases, the polymer chains have less freedom to move and slide past each other, resulting in higher resistance to deformation and higher shear storage modulus [42]. The larger crosslinking density and smaller pore size observed in the hydrogels are responsible for the greater shear storage modulus, according to Figure 8. 

**Figure 9:**
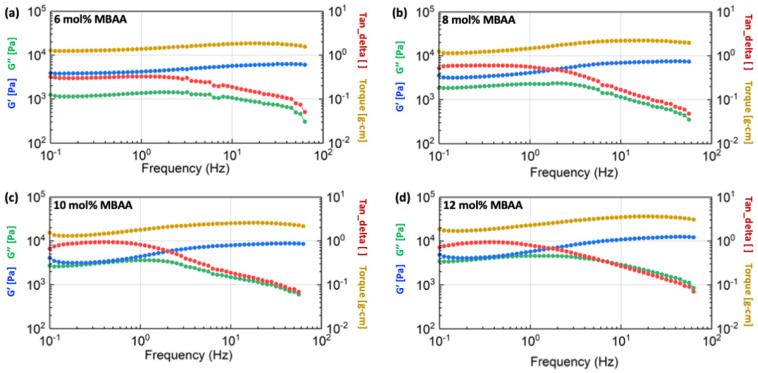
The rheological results of P(AAm-co-NaAMPS)-alginate-xanthan hydrogels with 2 mg/mL alginate concentration. The blue, green, yellow, and red curves represent shear storage modulus, shear loss modulus, torque, and tangent delta, respectively. The detailed MBAA concentrations in the hydrogel system are (**a**) 6 mol%, (**b**) 8 mol%, (**c**) 10 mol%, and (**d**) 12 mol%, respectively.

**Figure 10:**
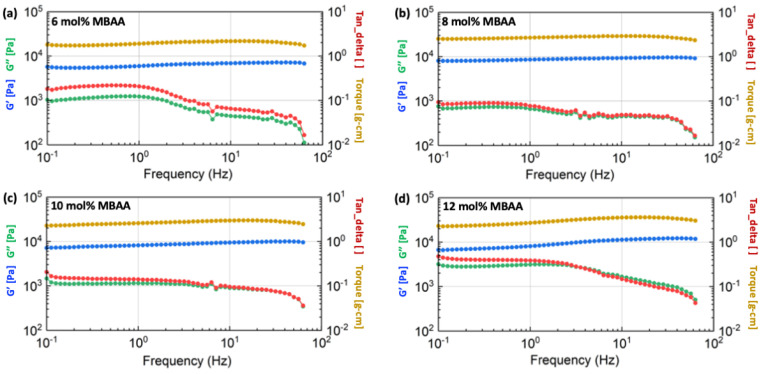
The rheological results of P(AAm-co-NaAMPS)-alginate-xanthan hydrogels with 4 mg/mL alginate concentration. The blue, green, yellow, and red curves represent shear storage modulus, shear loss modulus, torque, and tangent delta, respectively. The detailed MBAA concentrations in the hydrogel system are (**a**) 6 mol%, (**b**) 8 mol%, (**c**) 10 mol%, and (**d**) 12 mol%, respectively.

**Figure 11:**
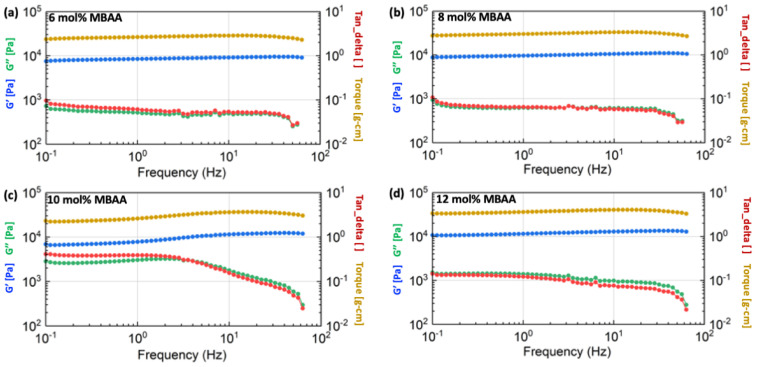
The rheological results of P(AAm-co-NaAMPS)-alginate-xanthan hydrogels with 6 mg/mL alginate concentration. The blue, green, yellow, and red curves represent shear storage modulus, shear loss modulus, torque, and tangent delta, respectively. The detailed MBAA concentrations in the hydrogel system are (**a**) 6 mol%, (**b**) 8 mol%, (**c**) 10 mol%, and (**d**) 12 mol%, respectively.

The detailed rheological results of the hydrogels with different compositions are shown in Figure 9, Figure 10 and Figure 11, including the information on shear storage modulus, shear loss modulus, tan delta, and torque.

Figure 9 demonstrates that the shear storage modulus (G’) of hydrogels with a 2 mg/mL alginate concentration increases as the concentration of the MBAA crosslinker is raised from 6 mol% to 12 mol%. This trend is similarly observed in the storage modulus of hydrogels with 4 mg/mL concentrations, as illustrated in Figure 10. However, as the alginate concentration is further increased to 6 mg/mL, the difference in shear storage modulus of hydrogels becomes less significant, as shown in Figure 11.

Thus, the energy storage capacity of the P(AAm-co-NaAMPS)-alginate-xanthan hydrogels under shear increases with the augmentation of MBAA. This trend becomes less significant when the alginate is raised to a higher concentration at 12 mg/mL.

#### 2.5.3. Cyclic Tensile Test Results of Hydrogels

**Figure 12:**
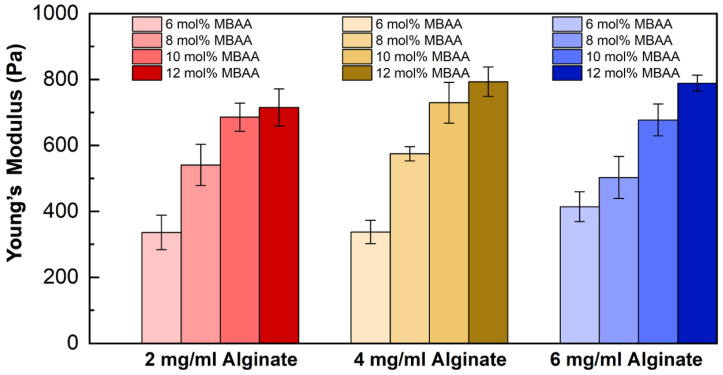
The Young’s modulus of the P(AAm-co-NaAMPS)-alginate-xanthan hydrogels with various compositions. The red, brown, and blue groups represent 2 mg/mL, 4 mg/mL, and 6 mg/mL of alginate, respectively.

The Young’s modulus of different P(AAm-co-NaAMPS)-alginate-xanthan hydrogels tested through cyclic tensile tests are shown in Figure 12. Within the red (first from the left) group, Young’s modulus doubles when the MBAA concentration reaches 8 mol%, and it even triples as the MBAA concentration continues to increase until it reaches twice the initial concentration. A similar trend can be observed in the brown (second) and blue (third) groups, where Young’s modulus of hydrogels increases significantly with higher MBAA content. 

As per Figure 12, MBAA plays an important role in increasing Young’s modulus of hydrogels. Across all alginate concentrations, the highest Young’s modulus was achieved at a 12 mol% concentration of MBAA. The increased crosslinking density and reduced pore size found in the hydrogels featuring 12 mol% MBAA contribute to the higher Young’s modulus exhibited by these hydrogels. As the MBAA crosslinker concentration rises, polymer chains in a hydrogel system become more extensively intra- and inter-molecular crosslinked, this increased interconnection in the hydrogel system restricts the movement of the polymer chains. Consequently, when a specific load is applied, the limited molecular mobility leads to reduced displacement within the hydrogel system and increased Young’s modulus of the hydrogels. The results can also be generalized and applied to other covalent-crosslinked hydrogel systems.

## 3. Conclusions

In this work, the effects of different factors—including MBAA, sodium alginate, and CaSO_4_—affect the properties of P(AAm-co-NaAMPS)-alginate-xanthan hydrogels were investigated. The obtained results can guide the synthesis of the hydrogels that are suitable for vascular graft applications as well as other tissue engineering applications. 

The increased sodium alginate content leads to increased equilibrium swelling ratios, greater viscosity in hydrogel precursor solutions, and reduced transparency. Introducing additional CaSO_4_ decreases the swelling ratio of a hydrogel system, which offsets the increased swelling ratio caused by the addition of more alginate. Increased MBAA in the hydrogel system enhances both the shear modulus and Young’s modulus while reducing the pore size and transparency of a hydrogel system. Overall, Hydrogel (6A12M) with 2.58 mg/mL CaSO_4_ was found to be the optimal candidate to use for the novel ePTFE–hydrogel vascular grafts that benefit from the reliable properties of ePTFE vascular grafts and favorable biological attributes of hydrogels, collectively.

## 4. Materials and Methods

### 4.1. Materials

In the chemically crosslinked copolymer system, two kinds of monomers, namely, acrylamide (AAm, CAS No. 79-06-1) and 2-acrylamido-2-methyl-1-propanesulfonic acid sodium salt solution (NaAMPS, CAS No. 5165-97-9), were purchased from Calbiochem (San Diego, CA, USA) and Sigma-Aldrich (St. Louis, MO, USA), respectively. The initiator of the copolymer hydrogel system is ammonium persulfonate (APS, CAS No. 7727-54-0), the crosslinker is N,N’-methylenebisacrylamide (MBAA, CAS No. 110-26-9), and the crosslinking accelerator in the hydrogel system is N,N,N’,N’-Tetramethylethylenediamine (TEMED, CAS No. 110-18-9), all purchased from Sigma-Aldrich. 

In the ionically crosslinked copolymer system, xanthan gum (XG, CAS No. 11138-66-2) was purchased from TCI America (Portland, OR, USA), sodium alginate (CAS No. 9005-38-3) was bought from Sigma-Aldrich, and calcium sulfate (CaSO_4_, CAS No. 7778-18-9) was purchased from Thermo Fisher Scientific (Waltham, MA, USA). 

### 4.2. Methods

To synthesize P(AAm-co-NaAMPS)-alginate-xanthan hydrogels, the method described in the previous research was employed [25]. First, aqueous precursor solutions of 0.5 mol% APS, 0.6 mol/L AAm, 0.4 mol/L NaAMPS, 8 mg/mL xanthan, along with varying amounts of MBAA and sodium alginate were prepared, thoroughly mixed, and degassed. The naming labels of various hydrogel samples and their corresponding MBAA and sodium alginate compositions are provided in Table 2. Next, a slurry of calcium sulfate and TEMED was prepared and injected into an annular mold defined by the ePTFE graft and a PTFE rod insert. Subsequently, the precursor solution was poured into the mold to be thoroughly mixed with the calcium sulfate slurry without introducing bubbles.

To thermally initiate the polymerization process, the molds with hydrogel precursor solutions were placed in an oven set at 65 °C for a duration of 2 h. Following this, the samples were left at room temperature overnight to ensure complete polymerization. Consequently, P(AAm-co-NaAMPS)-alginate-xanthan hydrogels with different compositions were successfully synthesized, as illustrated in Figure 1.

**Figure 13:**
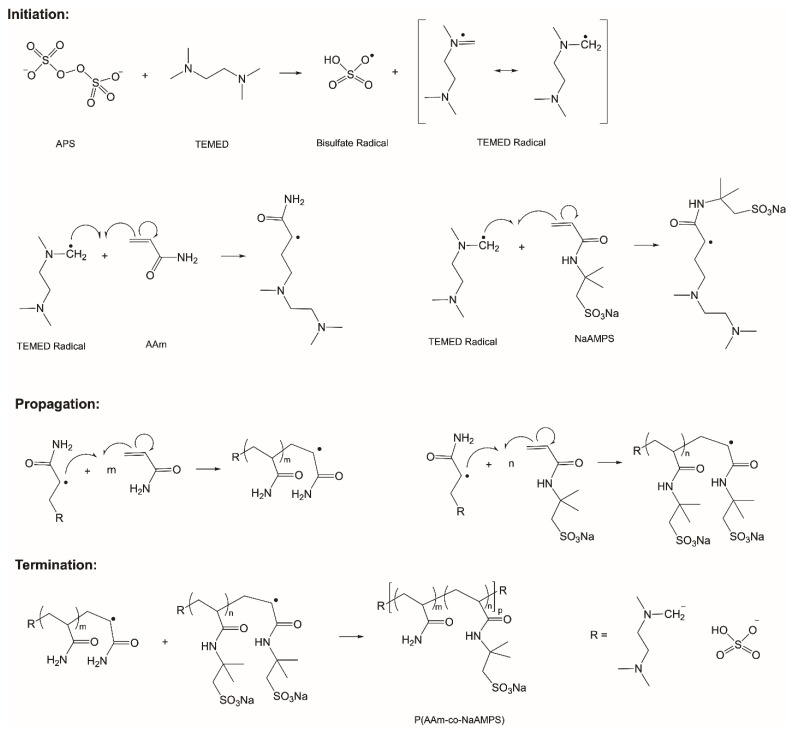
The initiation, propagation, and termination of the polymerization process in the chemically crosslinked P(AAm-co-NaAMPS) copolymer hydrogel systems. The black dots and arrows indicate the movement of electrons.

Two crosslinking methods were employed simultaneously to create the hydrogels: covalent crosslinking and ionic crosslinking. The ionic crosslinking hydrogels were formed through the combination of xanthan and alginate together with CaSO_4_, while the covalent crosslinking hydrogels were achieved through the synthesis of P(AAm-co-NaAMPS) copolymer. The P(AAm-co-NaAMPS) copolymer hydrogel synthesis reaction diagram presented in Figure 13 includes the initiation, propagation, and termination processes. While this study employs different material compositions of crosslinker and thickeners, detailed synthesis and fabrication procedures of the crosslinked ePTFE–hydrogel vascular graft systems can be found in references [25,43].

### 4.3. Characterizations

#### 4.3.1. Fourier Transform Infrared (FTIR) Analysis 

P(AAm-co-NaAMPS)-alginate-xanthan hydrogels with various compositions were initially frozen in the −70 °C freezer, then freeze-dried and subsequently pulverized into their respective powders for the analysis of functional groups in different hydrogels. These freeze-dried hydrogel powders were then finely ground and thoroughly mixed with 2.5% potassium bromide (KBr). Following this, the resulting ground freeze-dried hydrogel-KBr powders were compacted into circular discs to facilitate Fourier transform infrared (FTIR) testing using the transmission mode. FTIR spectra for these freeze-dried hydrogel powders were collected using a Nicolet iS50R Research FTIR Spectrometer within the 4000–400 cm^−1^ wavelength range.

#### 4.3.2. Scanning Electron Microscopy (SEM) Analysis

To investigate the microstructure of the hydrogels, the cross-section of the freeze-dried P(AAm-co-NaAMPS)-alginate-xanthan hydrogel samples were sputter-coated with a 10 nm gold layer using a Prep-Leica ACE600 sputter coater. Following sputter coating, the morphology and microstructure of the hydrogels were observed using a scanning electron microscope (SEM, JEOL NeoScope JCM-5000, Tokyo, Japan) operated at an accelerating voltage of 3 kV.

#### 4.3.3. Equilibrium Swelling Ratios

The hydrogel samples, each with a diameter of 12.45 mm and varying compositions, were immersed in Petri dishes filled with Dulbecco’s phosphate-buffered saline (DPBS) and left at room temperature for a week. After this immersion period, the hydrogels were removed from the Petri dishes, and any residual water droplets were gently removed using Kimwipes [44]. The swelling ratios (*SR*) of the hydrogels were calculated by

(1) $$\begin{array}{c} SR=\frac{W_{t}-W_{0}}{W_{0}}\times 100\% \end{array}$$

where *W*_0_ and *W_t_* correspond to the initial weight and the weight after soaking in DPBS of the hydrogels, respectively. Three samples were tested for each hydrogel composition.

#### 4.3.4. Mechanical Properties

##### Cyclic Tensile Test

To determine the Young’s modulus of the hydrogel systems, cyclic tensile tests were conducted using the Instron machine (Model 5967) to measure the Young’s modulus of the hydrogel samples with different compositions. The hydrogel samples were prepared following the ASTM D638-22 guideline for cyclic tensile tests [45], with three samples tested for each composition. The testing was carried out using a triangular waveform, ramping to the maximum strain of 2% and then returning to the initial displacement, all at a strain rate of 0.80 mm/mm-min for 5 cycles. 

##### Rheological Test

To investigate the viscosity of hydrogel precursor solutions and the rheological properties of hydrogel samples under shear, an ARES Rheometer from TA Instruments (New Castle, DE, USA) was employed, using a 25 mm parallel plate configuration for hydrogel testing and a cone-and-plate configuration for hydrogel precursor solution testing. 

The parallel-plate hydrogel samples for parallel plate configuration were carefully prepared, each possessing a diameter of 25 mm and a thickness of 2 mm. The rheological tests were performed at room temperature, with a 2 mm gap between the plates. The hydrogel precursor solutions were thoroughly mixed and degassed. Subsequently, the solution was poured onto the bottom plate, ensuring full coverage before conducting the rheology test using the cone-and-plate configuration.

The tests employed the frequency sweep method, with the frequency range from 0.1 Hz to 100 Hz for hydrogel testing and from 0.01 to 100 Hz for hydrogel precursor testing, maintaining a constant 1% strain. Three parallel experiments were conducted for each individual sample.

### 4.4. Statistical Analysis

One-way analysis of variance (ANOVA) was used for statistical analysis, where *p*-values less than 0.05 were considered statistically significant.

## Figures and Tables

**Table 1:** The average pore size of P(AAm-co-NaAMPS)-alginate-xanthan hydrogels.

	Pore Diameter (μm)			
	6 mol% MBAA	8 mol% MBAA	10 mol% MBAA	12 mol% MBAA
6 mg/mL Alginate	316.81 ± 70.09	189.58 ± 44.93	235.49 ± 63.47	139.78 ± 24.85
4 mg/mL Alginate	352.04 ± 86.36	261.03 ± 74.33	242.92 ± 42.37	205.08 ± 69.42
2 mg/mL Alginate	330.47 ± 71.75	241.28 ± 58.92	203.95 ± 64.43	155.97 ± 20.13

**Table 2:** Naming labels and the corresponding MBAA and alginate compositions of P(AAm-co-NaAMPS)-alginate-xanthan hydrogels.

Hydrogel Type	Alginate (mg/mL)	MBAA (mol%)
Hydrogel (2A6M)	2	6
Hydrogel (2A8M)		8
Hydrogel (2A10M)		10
Hydrogel (2A12M)		12
Hydrogel (4A6M)	4	6
Hydrogel (4A8M)		8
Hydrogel (4A10M)		10
Hydrogel (4A12M)		12
Hydrogel (6A6M)	6	6
Hydrogel (6A8M)		8
Hydrogel (6A10M)		10
Hydrogel (6A12M)		12

## Data Availability

The data presented in this study are openly available in article.
